# Targeting NAD^+^ metabolism: dual roles in cancer treatment

**DOI:** 10.3389/fimmu.2023.1269896

**Published:** 2023-12-05

**Authors:** Jiaxin Yong, Songqing Cai, Zhaolei Zeng

**Affiliations:** ^1^ State Key Laboratory of Oncology in South China, Guangdong Provincial Clinical Research Center for Cancer, Sun Yat-sen University Cancer Center, Guangzhou, China; ^2^ Research Unit of Precision Diagnosis and Treatment for Gastrointestinal Cancer, Chinese Academy of Medical Sciences, Guangzhou, China

**Keywords:** NAD+ metabolism, tumor microenvironment, cancer treatment, NAMPT inhibitor, CD38, cancer immunotherapy

## Abstract

Nicotinamide adenine dinucleotide (NAD^+^) is indispensable for various oxidation-reduction reactions in mammalian cells, particularly during energy production. Malignant cells increase the expression levels of NAD^+^ biosynthesis enzymes for rapid proliferation and biomass production. Furthermore, mounting proof has indicated that NAD-degrading enzymes (NADases) play a role in creating the immunosuppressive tumor microenvironment (TME). Interestingly, both inhibiting NAD^+^ synthesis and targeting NADase have positive implications for cancer treatment. Here we summarize the detrimental outcomes of increased NAD^+^ production, the functions of NAD^+^ metabolic enzymes in creating an immunosuppressive TME, and discuss the progress and clinical translational potential of inhibitors for NAD^+^ synthesis and therapies targeting NADase.

## 1 Introduction

NAD^+^ is necessary for numerous energy production-related oxidation-reduction reactions. To fulfill the need for high rates of growth and biomass generation, cancer cells undergo metabolic reprogramming in unfavorable conditions, such as hypoxia. Warburg effect, a representative type of metabolic reprogramming in tumor cells, refers to a metabolic shift to glycolysis from mitochondrial oxidative phosphorylation (OXPHOS). According to recent research, cells turn to aerobic glycolysis when there is an increasing demand for NAD^+^ (1). The research offered novel perspectives on aerobic glycolysis in rapid-proliferating cells. Furthermore, NAD^+^-consuming enzymes (NADases) directly or indirectly impact DNA repair and gene transcription (2, 3).

**Figure 1:**
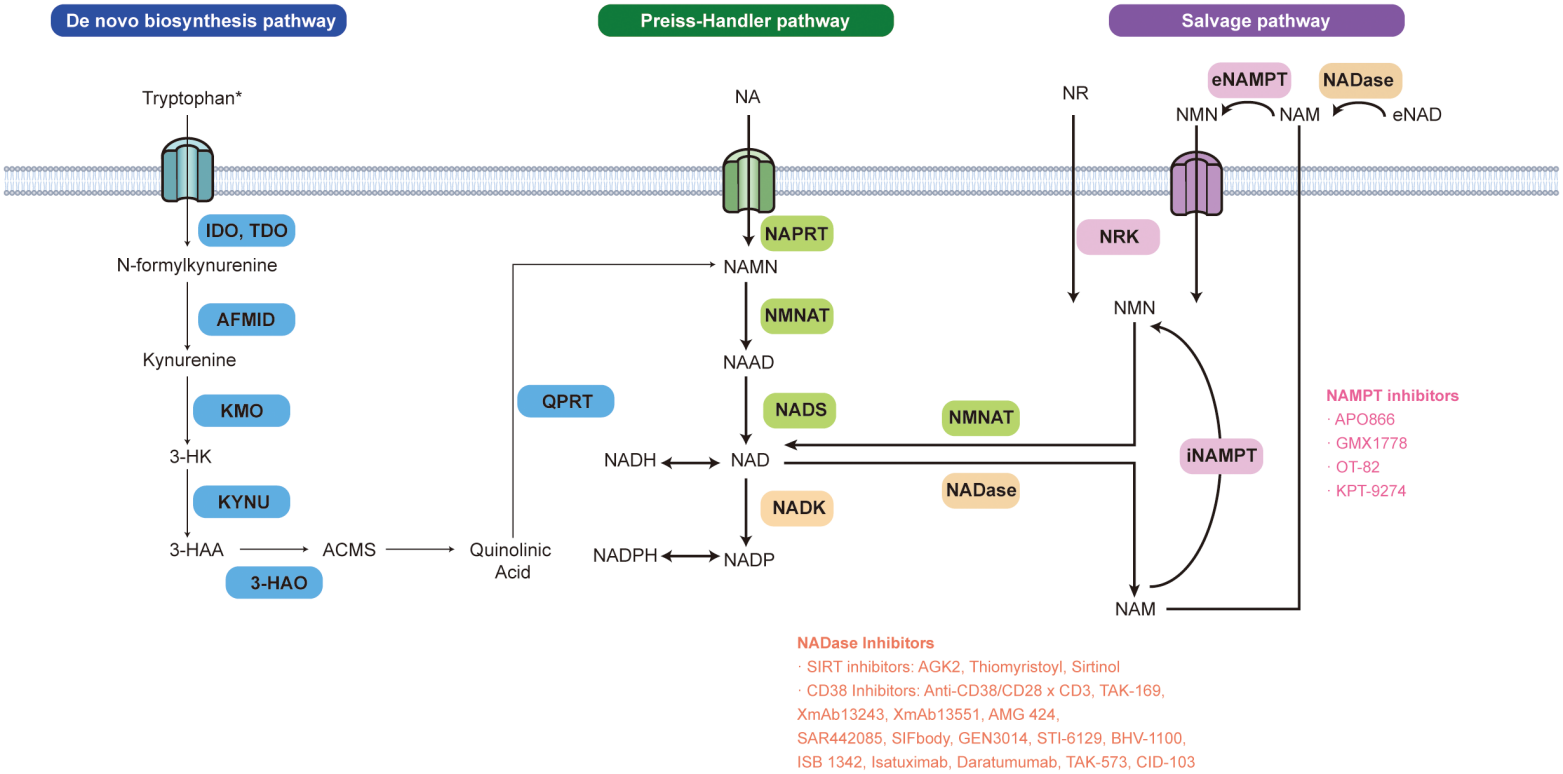
NAD^+^ biosynthesis pathways and inhibitors related to NAD^+^ metabolic enzymes. NAD^+^ can be produced via three pathways in mammalian cells: *de novo*, Preiss-Handler (PH), and the salvage pathway. The first rate-limiting step of the *de novo* NAD^+^ synthesis pathway is catalyzed by indole-2,3-dioxygenase 1,2 (IDO1,2) mainly expressed in the nervous system and tryptophan 2,3-dioxygenase (TDO) predominantly expressed in the liver. In this reaction, tryptophan is oxidized into the N-formyl kynurenine. The arylformamidase (AFMID) next catalyzes the conversion of N-formyl kynurenine to the immunosuppressive tryptophan metabolite kynurenine (Kyn). Kyn can be further metabolized to form quinolinic acid (QA), which is then decarboxylated by QA phosphoribosyl transferase (QPRT), to the nicotinic acid mononucleotide (NAMN) and ultimately to NAD^+^ via the PH pathway. NAD^+^ can also be made from metabolite recycling or the dietary uptake of NAD^+^ precursors (vitamin B3 and its derivatives) via the PH pathway or salvage pathway. Nicotinic acid (NA), the precursor of the PH pathway generates NAMN by nicotinic acid phosphoribosyltransferase (NAPRT). NAMN is transformed into nicotinic acid adenine dinucleotide (NAAD) by nicotinamide mononucleotide adenylyltransferases (NMNAT) The whole PH pathway ends up with NAD^+^ synthase (NADS) turning NAAD into NAD^+^. The NAD^+^ salvage pathway also interacts with the PH pathway in the human body. A gut microbial nicotinamidase (PncA) can convert ingested nicotinamide (NAM), one of the precursors of NAD^+^ salvage production, to NA which can enter the PH pathway. Besides dietary uptake, NADases such as CD38 and CD157, cleave NAD^+^ into NAM which is recycled and transformed into nicotinamide mononucleotide (NMN) by intracellular or extracellular nicotinamide phosphoribosyltransferase (NAMPT). Nicotinamide riboside (NR) generates NMN via nicotinamide riboside kinases 1 and 2 (NRK1,2) as well. Then NMN is transformed into NAD^+^ through NMNAT. *It is noted that most tissues, except the liver, don’t express the complete array of enzymes to synthesize NAD^+^ *de novo*.

In mammalian cells, NAD^+^ is mainly generated through three pathways: the *de novo* synthesis pathway, the Preiss-Handler (PH) synthesis pathway, and the salvage pathway. Notably, most tissues, except the liver, lack the full array of enzymes to synthesize NAD^+^ *de novo*, and thus, peripheral tissues utilize NAM derived from the liver to produce NAD^+^ through the salvage pathway (4). However, monocyte-derived macrophages (MDMs) can synthesize NAD^+^ from tryptophan, which delineates the polarization of macrophages and further regulates the innate immune response process of aging and inflammation (5). The most important enzymes involved in the PH pathway and salvage pathway are NAPRT (nicotinic acid phosphoribosyltransferase) and NAMPT (nicotinamide phosphoribosyltransferase), respectively. A recent analysis has shown that the tissue context mainly determines the type of NAD^+^ anabolic pathway activated in cancer. Because of their dependence on NAD^+^ production pathways, tumors can be divided into PH-amplified tumors, with a high frequency of tumors showing NAPRT amplification, and salvage-dependent tumors, which undergo NAMPT enhancer remodeling (6). Moreover, there have been multiple categories of NADases discovered, which encompass deacetylases (sirtuins), glycohydrolases (SARM1, CD38, CD157), and poly (ADP-ribose) polymerases (PARPs). The byproduct of all these proteins is NAM, which can be recycled in the salvage pathway to produce NAD^+^ by NAMPT. The synthesis and degradation of NAD^+^ in tumor cells is a highly dynamic process (**Figure 1**). Tumorigenesis and cancer progression have been strongly linked to the upregulation of NAD^+^ synthesis and dysregulation of NADase, as indicated by a growing number of reviews (7–12).

Interestingly, inhibiting NAD^+^ synthesis and targeting NADase have shown positive implications for the treatment of cancer. This may be due to the difference in the main roles played by NAD^+^ synthetase and degrading enzymes. Enzymes involved in NAD^+^ synthesis mainly affect tumor internal biological behaviors, while NAD-degrading enzymes participate in the immunosuppressive tumor microenvironment. Here we summarize the detrimental outcomes of increased NAD^+^ production, the functions of NAD^+^ metabolic enzymes in creating an immunosuppressive TME, and discuss the progress and clinical translational potential of inhibitors for NAD^+^ synthesis and therapies targeting NADase.

## 2 NAD^+^ anabolism dysregulation and targeted therapy strategies in cancer

Since NAD^+^ is essential for mediating redox reactions in various metabolic pathways, malignant cells upregulate NAD^+^ biosynthesis enzyme genes to satisfy the high demands of proliferation and biomass production. A recent analysis has shown that healthy tissues from which NAD^+^ biosynthetic enzymes were derived determine whether the NAD^+^ salvage pathway is activated or the PH synthesis pathway is changed (6).

The expression of NAMPT is increased in urothelial carcinoma, breast cancer, stomach cancer, esophageal cancer, colorectal cancer (CRC), ovarian cancer, prostate cancer, glioblastoma, and melanoma. High expression levels of NAMPT are associated with unfavorable overall survival, advanced TNM stage, vascular invasion, and invasion depth (13–16). Furthermore, NAMPT is localized both intracellularly and extracellularly. Extracellular NAMPT (eNAMPT), is also known as visfatin or pre-B-cell colony-enhancing factor. Elevated serum eNAMPT levels indicate poor prognosis (17–21).

According to the upregulated NAD^+^ synthesis pathway, cancer cells can be divided into PH-amplified tumors, many of which show NAPRT amplification, and salvage-dependent tumors, which are characterized by NAMPT enhancer remodeling (6). PH-amplified cancers include ovarian cancer (22), prostate cancer, and pancreatic cancers (23), which are influenced by the increased expression of NAPRT (13–16, 24). In patients with CRC, a high level of NAPRT is associated with poor prognosis. Furthermore, CRC tissues have shown a correlation with NAPRT and NAMPT (14). On the other hand, gastric, colorectal, and renal carcinoma, along with various leukemia, have shown a low expression level of NAPRT (25). Mechanistically, NAPRT epigenetic expression is inhibited in certain tumors due to the methylation of its promoter (14). The occurrence of gene mutation, amplification, and methylation in NAPRT is more frequent than NAMPT (14, 25). In summary, NAPRT is a critical counterpart to NAMPT in NAD^+^ synthesis, but the role played by NAPRT is possibly underestimated and is rarely studied. The primary focus of most studies has been on the correlation between NAPRT and susceptibility to NAMPT inhibitors.

### 2.1 Metabolic reprogramming and oxidative stress

Intracellular NAMPT (iNAMPT) plays a pivotal role in the metabolism reprogramming of cancer cells. iNAMPT inhibition leads to impaired glycolytic flux, lactate production, and maximum mitochondrial respiratory capacity, eventually promoting energy abrogation (26). Inhibition of iNAMPT also impacts various metabolic pathways, such as the pathways for serine biosynthesis and one-carbon metabolism (27), amino acid metabolism (28), pentose phosphate pathway (29), lipid metabolism (30), purine and pyrimidine metabolism (28). Inhibiting NAMPT to suppress NAD^+^ production is a successful approach for modifying cancer cell metabolism and decreasing ATP levels (31). Likewise, NAPRT has a crucial function in controlling cellular levels of NAD^+^, OXPHOS, energy levels, protein production, and cellular dimensions. It is worth mentioning that the inhibition of NAPRT decreases OXPHOS in cancer cells that overexpress NAPRT and makes them more sensitive to NAMPT inhibitors (NAMPTi).

Oxidative stress results from an imbalance between reactive oxygen species (ROS) and cellular antioxidant capacity. Studies have demonstrated that iNAMPT enhances the antioxidant potential of cancer cells while reducing the harmful consequences of excessive accumulation of ROS (29, 32). In contrast, depletion, or inhibition of NAMPT resulted in antitumor effects by elevating levels of ROS in non-small cell lung cancer (NSCLC) (33), glioblastoma (34), prostate cancer (35), breast cancer (29), and colon cancer (32). In leukemia, NAMPTi induced ROS production, triggering cell death through mitochondria dysfunction and ATP loss (36).

Taken together, these discoveries offer a rationale for focusing on NAMPT/NAPRT as an innovative approach to trigger metabolic impairment resulting in cancer cell death.

### 2.2 The epithelial-mesenchymal transition (EMT) and cell stemness

Although cancer stem cells (CSCs) only constitute a small subpopulation with self-renewal properties, they are crucial not only to tumorigenesis but also to metastasis and therapy resistance. NAMPT has been described to enhance tumor aggressiveness by modulating the EMT and supporting cancer cell stemness (37, 38). NAMPT has been described to enhance tumor aggressiveness by modulating the EMT and supporting cancer cell stemness (37, 38). Importantly, the role of NAMPT in promoting EMT does not depend on its enzymatic activity (39). Visfatin/eNAMPT also induces EMT to promote cancer cell migration (39, 40). However, pharmacological NAMPT inhibition decreases the expression of SIRT1 and reverses the SIRT1−mediated EMT to inhibit the cell capacity for invasion and metastasis (41). Furthermore, there has been a recent report indicating a correlation between NAMPT activity and the characteristics of stemness in leukemia (42), glioma (43), colon cancer (44), and breast cancers (39).

NAMPTi decreases NAD^+^ levels and the self-renewal capacity of CSCs and inhibits tumorigenicity in glioblastoma (45). NAMPT suppression reversed the capability of cancer cells to dedifferentiate (46) and potentially decreased the CSC load in metastatic prostate cancer (47).

Mechanistically, NAMPT may modulate SIRT1 and PARP1 activity to regulate stem cell signaling pathways, thus influencing the EMT and tumor cell dedifferentiation (44). Hence, exploring the impact of NAMPT on the EMT and stemness in cancer cells can pave the way for the clinical application of NAMPT inhibitors, PARP inhibitors, and SIRT inhibitors.

### 2.3 Interactions with oncogenic signaling pathways

Extensive research shows that NAMPT engages in regulating oncogenic signaling pathways. NAMPT upregulation has been reported to be associated with the phosphorylation of AKT (48). NAMPT inhibition suppressed N-MYC expression and reduced the phosphorylation levels of AKT and GSK3 (49). Similarly, NAMPTi decreased the dephosphorylation rate of the EGFR, ERK1/2, MEK1/2, and Akt in NSCLC (50). AKT/PI3K and ERK/MAPK activation by visfatin/eNAMPT promote proliferation and prevent apoptosis in breast cancer, while AKT and ERK1/2 inhibition abrogated these effects (51). In addition, combining NAMPTi with MAPK inhibitors synergistically promoted cell death (52).

In addition, a connection between NAMPT and mTOR activation has been shown in various cancers. NAMPT inhibition suppressed mTOR signaling in multiple myeloma cells (52), hepatocarcinoma cells (53), and pancreatic cancer cells (54). mTOR inactivation induced by NAMPT inhibition may contribute to autophagic death and apoptosis, as reported for adult T-cell leukemia cells (55). Moreover, the effect of decreasing cell viability was more pronounced when NAMPT inhibitors were combined with an AMPK activator and an mTOR inhibitor (54). Further studies are needed to shed light on the mechanisms of action and to verify the potential effectiveness of combinations of NAMPTis and other targeted drugs against cancer.

### 2.4 Resistance to BRAF inhibitors and chemotherapy

Multiple selective BRAF inhibitors (BRAFis) have been used in combination with MEK inhibitors for the treatment of BRAF-mutated metastatic melanoma (56). However, the clinical benefits of using BRAFis have been limited by drug resistance. Several studies have revealed that increased NAD^+^ levels and high NAMPT expression were related to the development of drug resistance to BRAFis (57–59). High levels of both iNAMPT and visfatin/eNAMPT were expressed in BRAFi-resistant melanoma cells, while transcriptional downregulation of NAMPT was reported in BRAFi-sensitive cells (57–59). Furthermore, overexpressing NAMPT necessarily and sufficiently recapitulated the BRAFi-resistant phenotype plasticity in MM (59, 60). Nevertheless, pharmacological NAMPT inhibition induced energy abrogation and apoptosis to suppress cell growth in BRAFi-resistant MM cells (57). Thus, NAMPT may become an actionable target in BRAFi-resistant MM patients.

### 2.5 The regulation of DNA damage repair and sirtuin function

PARPs and sirtuins are NAD^+^-dependent consuming enzymes that are critical for cellular NAD^+^ degradation. PARPs participate mainly in DNA repair, while SIRTs constitute a family of deacetylase proteins. Given that NAMPT is involved in the generation of NAD^+^, NAMPT has an impact on DNA damage repair and sirtuin function.

A functional relationship between NAMPT and PARPs has been proposed based on evidence showing that NAMPTi increased DNA damage because of the loss of PARP activity in Ewing sarcoma and pediatric acute lymphoblastic leukemia (61, 62). The synergistic combination of NAMPT and PARP inhibition increased the inhibitory effect on tumor cells (63, 64). However, other evidence has suggested that PARP1 activation enhanced the antitumor effects of NAMPTi, suggesting a complex and controversial relationship between PARP and NAMPT (36, 65). PARP1 is the best-characterized PARP that is actively involved in chromatin organization and the DNA repair pathway (12). NAMPT is located in both the nucleus and cytoplasm. It has been recently shown that NAMPT and GAPDH form a stable complex that mediates the translocation of NAMPT. NAMPT translocation contributes to the nuclear NAD^+^ pool, which increases PARP1 activity and response to stressors, such as DNA damage induced by UV light (66, 67). All these studies lay the groundwork for the preclinical development of NAMPTis with modulators of PARP activity.

SIRTs are a group of deacetylase proteins that make use of NAD^+^ as a coenzyme. Notably, SIRTs are upregulated along with NAMPT in several solid cancers, including breast, prostate, gastric, colorectal, liver, and pancreatic cancers (21). Inhibition of NAMPT led to decreased expression and activity of SIRT1 (41, 68), SIRT2 (69), and SIRT3 (70). Several studies have reported that c-MYC increased the expression and activity of SIRT1 mediated via transcriptional activation of NAMPT to drive tumor cell proliferation and progression (71–73). Moreover, in addition to iNAMPT, visfatin/eNAMPT regulated the expression levels of SIRT1 and P53 to promote cell proliferation (74, 75).

Interestingly, it has been noted that the anticancer effects of NAMPTis in pancreatic cancer are not influenced by either SIRT1 or PARP1, indicating that the impact of NAMPT on PARP1 or sirtuins may vary depending on the type of cancer. Determining the cancer types that are more responsive to NAMPTis is crucial for the future clinical translation of NAMPTis.

## 3 Enzymes in NAD^+^ metabolism immunomodulate the tumor microenvironment

### 3.1 NAMPT and NAPRT

Notably, in contrast to its intracellular form, visfatin/eNAMPT can function as an immunomodulatory cytokine (76, 77). eNAMPT is present in the extracellular space and facilitates strong NF-κB transcriptional stimulation through its interaction with Toll-like receptor 4 (TLR4) (78). eNAMPT induces the release of TNF-α, IL-1α, IL-1β, IL-6, and IL-8, and enhances the expression of the costimulatory molecules CD40, CD54, and CD80 (79–81). Interestingly, extracellular NAPRT (eNAPRT) is structurally and functionally similar to eNAMPT. By binding to TLR4, eNAPRT activates the inflammasome and NF-κB pathway, leading to the release of inflammatory cytokines. Moreover, eNAPRT induces the differentiation of monocytes into macrophages through the enhancement of transcription and secretion of macrophage colony-stimulating factor (M-CSF/CSF1). eNAMPT, when bound to C-C chemokine receptor type 5 as an antagonist, hinders Rantes-dependent calcium signaling in melanoma. The cytokine-like properties appear to be unrelated to its enzymatic function (82).

So far, it has been demonstrated that NAMPT affects myeloid cells as well in the TME. eNAMPT-primed M2 macrophages support the survival of leukemic cells and suppress T-cell responses by increasing the activity of the PD-1/PD-L1 axis. Additionally, NAMPT exerts its effects on myeloid-derived suppressor cells (MDSCs) (83). NAMPT regulates the mobilization of MDSCs and boosts their generation of inhibitory nitric oxide. Inhibiting NAMPT pharmacologically leads to synergistic effects with an anti-PD-1 antibody and releases MDSC restraints on antitumor immunity (84, 85). Moreover, NAMPT, which is downstream of G-CSF (CSF3) receptor signaling, promotes the tumorigenic conversion of tumor-associated neutrophils (TANs), enhancing angiogenesis via SIRT1 activity. TANs from head-and-neck cancer and melanoma patients have been characterized by high NAMPT expression (86).

The mechanisms behind the immunomodulation effects of NAMPT on TME are still being studied. Further studies are needed to shed light on the role of NAMPT in establishing the immunosuppressive tumor microenvironment.

### 3.2 SIRTs

SIRTs play contradictory and complicated roles in regulating cancer cell growth and proliferation. SIRTs may act as promoters or suppressors in different kinds of cancers (87). Recently, SIRTs have also been reported to play potential roles in antitumor immunity. In an ovarian cancer model, artesunate, a medication used to treat malaria, induced Th1 cell differentiation from CD4+ T cells and showed enhanced proapoptotic effects on ovarian cancer cells by upregulating miR-142 and suppressing SIRT1 levels (88). Furthermore, the overexpression of SIRT2 in TILs of cancer patients has been linked to a negative outcome in terms of their response to immunotherapy. In contrast, SIRT2 inhibition during T-cell activation triggers the hyperacetylation of various metabolic enzymes, resulting in elevated aerobic glycolysis and OXPHOS. In other words, the absence of SIRT2 resulted in hyperreactive T cells with enhanced antitumor activity that overcame the immunological and metabolic barriers within suppressive tumor microenvironments (89). Additionally, there have been reports indicating that SIRT5 has a regulatory function in the differentiation of CD4+ regulatory T (Treg) cells and T helper 1 (Th1) cells during colorectal tumorigenesis (90). SIRT6 increased the levels of Ca^2+^-mobilizing second messengers and enhanced the expression of proinflammatory cytokines in pancreatic cancer, linking SIRT6 with a cancer cell proinflammatory phenotype and migratory propensity (91). Finally, SIRT7 inhibited the expression of PD-L1 by forming a complex with MEF2D, suggesting that strategies to modulate the activity of SIRT7 may potentially enhance the efficacy of immunotherapies in hepatocellular carcinoma (92). Further investigation is needed to explore the role of SIRT in the TME, despite some previous studies on the regulatory impact of SIRTs on T-cell metabolism and function (93).

### 3.3 Glycohydrolases: CD38, CD157 and SARM1

CD38, a transmembrane protein, is widely expressed in endothelial cells (94), fibroblasts (95), smooth muscle cells (96), and various immune cells (97). The main biological role of CD38 is hydrolyzing β-NAD^+^ into NAM and ADPR. It shows poor ADP-ribosyl cyclase activity, thereby inefficiently producing a minor amount of cyclic ADPR (cADPR). Interestingly, it catalyzes the hydrolysis of cADPR to ADP-ribose (ADPR) (98–100). Both ADPR and cADPR serve as second messengers in the modulation of intracellular calcium signaling (101). CD38/ADPR/Ca^2+^ signaling immunomodulates neutrophil chemotaxis, lymphocyte proliferation, and T-cell activation (102, 103). On the other hand, in the TME, CD38 participates in the production of noncranial extracellular adenosine (eADO), relying on NAD^+^ as a substrate to generate ADPR. CD203a processes ADPR into extracellular AMP (eAMP). Then, CD73 catalyzes the hydrolysis of AMP to generate extracellular ADO (eADO). Through its purinergic receptor binding, especially A_2_A and A_2_B, adenosine is important in the suppression of both innate and adaptive immune responses. Adenosine restrains the antitumor immune response by inhibiting the anticancer activity of CD8^+^ T cells and NK cells and recruiting MDSCs and Treg cells. ADO/A_2_A signaling suppresses T cells producing IFN γ, TNF, granzymes, and perforin (104). There has been speculation that CD38, by participating in the regulation of NAD^+^ and adenosine homeostasis, could potentially function as an immune checkpoint (104, 105).

CD157 is generated in two forms: in a soluble form (106) and as a glycosylphosphatidylinositol (GPI)-anchored glycoprotein. Similar to its paralog CD38, CD157 shows both glycohydrolase and weak ADP-ribosyl cyclase functions (107–109). It converts β-NAD^+^ into NAM, substantial ADPR, and a small amount of cADPR. Despite being less efficient than CD38, the ADP-ribosyl cyclase function of CD157 significantly improves under acidic conditions and with the presence of Zn^2+^ and Mn^2+^ (108, 109). Considering its role in ADPR generation, CD157 is potentially involved in the CD38/CD157/CD203a/CD73 adenosinergic signaling, increasing ADO levels to favor immunosuppressive TME formation (110). Notably, although it lacks intracellular domains, CD157 in the GPI-anchored form functions as a receptor. The interaction between CD157 and fibronectin plays a crucial role in the formation of a complex with integrins, facilitating the creation of a connected network that transmits signals both inside and outside the cell. This process greatly enhances cell adhesion to the extracellular matrix, promotes cell migration, and ensures cell survival (111, 112). The role played by CD157 as a receptor needs to be further analyzed, particularly its role in regulating the immunosuppressive TME, which has been largely uncharacterized.

SARM1 was initially classified as a negative regulator of TRIF-dependent TLR signaling (113). SARM is mainly expressed in neurons and T lymphocytes, mediating cell death and promoting the neuronal inflammatory response (114–117). SARM1 has recently been designated a new class of NAD^+^ glycohydrolase in axonal degeneration (118–120). The intrinsic NADase activity of SARM1 depends on the Toll/interleukin receptor domain converting NAD^+^ to ADPR, cADPR, and NAM, with NAM inhibiting the enzyme in a feedback loop. Notably, NAM also suppresses other NAD^+^-consuming enzymes (121, 122). Furthermore, its enzymatic activity is essential for bacterial innate immunity (123). However, in contrast to its undisputed pivotal role in axonal degeneration, the immunoregulatory role of NAM remains unclear.

## 4 The development of drugs targeting NAD^+^ metabolism

### 4.1 Targeting NAD^+^ synthesis

Considering the critical role played by NAMPT in the NAD^+^ salvage pathway, an increasing number of pharmacological NAMPTis have been developed; these include specific NAMPT inhibitors (APO866, GMX1778, GMX1777, and OT-82) and dual NAMPT inhibitors (KPT-9274). Here, we summarize the most extensively studied NAMPTi and describe its antitumor effects *in vitro* and *in vivo*, as well as progress in its clinical applications (124, 125) (**Tables 1**, **2**).

**Table 1:** Preclinical studies of NAMPT inhibitors in cancer.

Compound	Cancer	IC50 for tumor cells	IC50 for normal cell	Effects *in Vitro*	Effects *in Vivo*	Reference
APO866	Hematologic malignancies	0.09 nM ~ 27.2 nM (96 h)	<10 nM (normal hematopoietic progenitor cells from mobilized peripheral blood, 96 h)	- Reduces intracellular NAD^+^ and ATP levels -Induces mitochondrial dysfunction and triggers autophagy - Has an inhibitory effect on hematologic tumor cell clonogenicity	-Abrogates tumor growth, prevents tumor development, and significantly prolongs mouse survival times	(126)
APO866	Glioblastoma	8.5 nM (C6 glioblastoma cells, 72 h)	Not available	- Reduces intracellular NAD^+^ levels and cell viability -Inhibits proliferation and induces G2/M cell-cycle arrest	Not available	(127)
GMX1778	Lung cancer	32.3 nM (A549 cells, 72 h) 49.8 nM (H226 cells, 72 h) 36.1 nM (H460 cells, 72 h) 1.1 nM (DMS-114 cells, 72 h) 0.8 nM (NYH cells, 72 h) 1.9 nM (SHP77 cells, 72 h)	Not available	-Induces NAD^+^ depletion and results in cancer cell death	-Coadministration with NA widens the therapeutic index of GMX1777 effectiveness in NAPRT1-deficient tumors	(128)
GMX1778	Breast cancer	31.0 ± 24.0 nM (MCF-7 cells, 96 h)	5650 ± 212 nM (human umbilical vein endothelial cells, 72 h)	-Exhibits potent cytotoxic effects on human breast cancer cells	-Reduces tumor volumes without including a significant change in body weight	(129)
OT-82	Pediatric acute lymphoblastic leukemia (ALL)	0.2~4.0 nM (14 acute leukemia cell lines, 72 h)	Not available	-Potently decreases the viability of leukemia cells -Reduces cellular NAD^+^ levels and induces apoptosis -	-Induces significant leukemia regression -Enhances the activity of established drugs used in the treatment of pediatric high-risk ALL	(130)
OT-82	Hematopoietic (HP) cancer, breast, prostate, pancreatic, cervical, ovarian, colon and lung carcinomas, melanoma and glioblastoma	2.89 ± 0.47 nM (HP cancer cell lines, 72 h) 13.03 ± 2.94 nM (non-HP cancer cell lines, 72 h)	62.69 ± 18.20 nM (normal human bone marrow mononuclear cells, 72 h)	-Strongly inhibits hematopoietic malignancy progression	-Decreases tumor growth and increases median survival times -Optimization of OT-82 dosing and dietary niacin further expands the OT-82 therapeutic index	(131)
KPT-9274	Non-Hodgkin’s lymphomas	95.17 nM (WSU-DLCL2 cells, 72 h) 13.9 nM (WSU-FSCCL, 72 h)	1836 nM (peripheral blood mononuclear cells (PBMCs) isolated from the blood of a healthy, nonsmoking donor, 72 h)	-Induces apoptosis and cell metabolic collapse in WSU-DLCL2 and WSU-FSCCL cell lines -Enhances the efficacy of chemotherapeutic agents	-Reduces tumor volumes without inducing a significant change in body weight -Inhibits pak4 and activates caspase 9 -Prolongs animal survival times	(132)
KPT-9274	Renal cell carcinoma	600 nM (Caki-1 cells, 72 h) 570 nM (786-0 cells, 72 h)	1,300 nM (normal primary renal proximal tubular epithelial cells, 72 h)	-Causes NAD^+^ depletion -Attenuates cell viability, invasion, and migration	-Decreases tumor growth with no significant weight loss -Decreases expression of PAK4, cyclin D1, and SIRT1	(133)
KPT-9274	Renal cell carcinoma	∼500 nM (pancreatic ductal adenocarcinoma cell lines, 72 h)	> 5 μM (human pancreatic ductal epithelial cells, 72 h)	-Induces apoptosis, cell-cycle arrest and suppresses migration -Overcomes stemness -Synergizes with gemcitabine and oxaliplatin	-Significantly inhibits tumor growth with induces no body weight loss or tumor rebounding with used with a single NAMPTi and in combination with gemcitabine -Suppresses highly resistant CSC–derived PDAC	(134)
KPT-9274	Acute myeloid leukemia	27 to 215 nM, 48 h	Not available	-Diminishes NAD^+^ levels and cellular respiration, leading to cell death	-Prolongs overall survival and suppresses disease progression	(135)
KPT-9274	B-cell acute lymphoblastic leukemia (B-ALL)	<35 nM, 72h	Not available	-Reduces the cellular NAD^+^ level -Inhibits B-ALL cell growth and induces apoptotic cell death	-Effectively suppresses leukemia progression and prolongs survival without inducing body weight changes	(136)
KPT-9274	Pancreatic Neuroendocrine Tumors (PNET)	77.29 nM (BON-1 cells, 72 h) ~140.6 nM (QGP-1 cells, 72 h)	Not available	-Decreases PNET cell survival and growth -Synergizes with everolimus	-Significantly reduces tumor growth -Suppresses the expression level of anti-apoptotic markers and induces pro-apoptotic marker expression	(137)
KPT-9274	Multiple myeloma	<6 μM (23 myeloma cell lines, 48 h)	<5μM (PBMCs, 48 h)	-Inhibits cell growth and reduces survival time in a large panel of multiple myeloma cell lines -Suppresses bone marrow microenvironment-mediated effects	-Induces tumor cell death	(138)
KPT-9274	Ewing sarcoma	<100 nM(A673, CHLA-10, TC32, 72 h)	Not available	-Reduces tumor cell viability, migratory, and invasiveness	- Reduces tumor growth -Significantly inhibits primary and metastatic tumor formation	(139)
KPT-9274	Rhabdomyosarcoma	40~80 nM (multiple cell lines, 72 h)	>600 nM (normal skeletal muscle myoblasts, 72 h)	-Attenuates the acquisition of molecular signatures involved in cell cycle and metastatic progression	-Leads to regression of tumor growth and metastatic progression without inducing significant weight loss	(140)

**Table 2:** Clinical trials with NAMPTis used to treat cancer.

Type	Inhibitor	Tumor type	Phase	Clinical trial Identifier	Recruitment status
Specific NAMPT Inhibitors	OT-82	Lymphoma	Phase 1	NCT03921879	Unknown
	GMX1777	Solid Tumors and Lymphomas	Phase 1	NCT00457574	Withdrawn (study terminated prematurely due to financial constraints.)
	GMX1777+ Temozolomide	Metastatic Melanoma	Phase 1/2	NCT00724841	Terminated (study terminated prematurely)
	CHS-828	Solid Tumor	Phase 1	NCT00003979	Withdrawn
	APO866	Melanoma	Phase 2	NCT00432107	Completed
	APO866	Cutaneous T-cell Lymphoma	Phase 2	NCT00431912	Completed
	APO866	B-Cell Chronic Lymphocytic Leukemia	Phase 1/2	NCT00435084	Completed
Dual Inhibitor	ATG-019(another name is KPT-9274)	Advanced Solid Tumors or Non-Hodgkin’s Lymphoma	Phase 1	NCT04281420	Recruiting
	KPT-9274	Advanced Solid Malignancies or Non-Hodgkin’s Lymphoma (NHL)	Phase 1	NCT02702492	Terminated (sponsor decision)
	KPT-9274	Acute Myeloid Leukemia	Phase 1	NCT04914845	Recruiting

APO866 (FK866), was the initial compound identified as an NAMPTi. It inhibits cancer cell proliferation and induces tumor regression (126, 127). GMX1778 (CHS-828) and OT-82 exert effects similar to each other, highlighting their beneficial effects in the treatment of solid cancers and hematological malignancies (128–131). Interestingly, KPT-9274 (also called ATG-019), which targets both NAMPT and serine/threonine p21-activated kinase 4 (PAK4), also exerts potent anticancer effects (132–140). A Phase I clinical trial is currently enrolling patients diagnosed with lymphoma or solid tumors to assess the efficacy of PAK4 inhibition.

Despite promising results in preclinical studies, NAMPTi monotherapy led to few objective tumor responses in Phase I/II clinical trials (141–145). These outcomes may indicate that before NAMPTi application, biomarkers may be needed to select patients who may benefit from NAMPT inhibitors. Indeed, due to their dependency on the NAD^+^ salvage pathway, salvage-dependent cancers with NAPRT deficiency are sensitive to treatment with NAMPT inhibitors (128, 146). However, PH-dependent cancers with upregulated NAPRT expression are resistant to NAMPT inhibitors (22). An increasing number of studies show that tumors that rely on NAPRT for NAD^+^ synthesis are initially resistant to NAMPT inhibitors but become sensitive to NAMPT inhibitors after NAPRT downregulation, illustrating the need for NAPRT inhibitor development (22, 147, 148). Unfortunately, very few NAPRT inhibitors have been reported to date. Among the few NAPRT inhibitors, 2-Hydroxynicotinic acid (2-HNA) can sensitize PH-amplified pancreatic and ovarian cancer cells to NAMPT inhibitors. Other compounds with NAPRT-inhibitory activity include several nonsteroidal anti-inflammatory compounds (149–151). Recently, several new small-molecule NAPRT inhibitors (NAPRTis) have been identified in silico. These NAPRTis administered with the NAMPTi FK866 exerted a synergistic effect, suggesting the therapeutic potential of these compounds (152, 153). However, more studies are required to verify the efficacy of these NAPRT inhibitors.

In addition, to overcome the limitations of available NAMPTis, targets for drug development such as antibody-drug conjugates (ADCs), dual-target inhibitors, and proteolysis-targeting chimera (PROTAC) technology may increase the efficacy and sensitivity of NAMPT inhibitors. For instance, based on PROTAC technology, the NAMPT PROTAC A7 inhibited tumor-infiltrating MDSCs and boosted antitumor efficacy by degrading both iNAMPT and eNAMPT (85). Similarly pharmacological NAMPT inhibitors administered with anti-PD-1 antibodies exhibited synergy and released MDSC suppression to induce antitumor immune responses (84). All of these studies indicate that NAMPTis are promising anticancer drug candidates when used in combination with immunotherapy to enhance their effects.

### 4.2 Targeting NADase

#### 4.2.1 SIRTs

SIRTs play contradictory and complicated roles in the regulation of cancer cell growth and proliferation. SIRTs may function as promoters or suppressors in different kinds of cancers (87). SIRTs, both activators and inhibitors, have been reported to exert anticancer effects. As mentioned above, SIRTs participate in the modulation of the TME. In this review, we focus mainly on the roles of SIRT actions in antitumor immunity. SIRT inhibitors may improve anticancer immunotherapy (**Table 3**). In particular, SIRT7 disruption enhanced the efficacy of PD-1 blockade therapy in hepatocellular carcinoma cells. Unfortunately, since no specific SIRT7 inhibitors have been developed, researchers use CRISPR/Cas9 to knock out SIRT7 to mimic the effects of SIRT7 inhibitors. We firmly believe that other SIRT modulators may play roles in immunotherapy and can increase the efficacy of immunotherapy. However, more research is still needed to further understand the roles played by SIRTs in antitumor immunity and develop more specific SIRT inhibitors.

**Table 3:** Studies on the inhibition mediated by SIRTs in anticancer immunity.

Name	Target	Roles	Effects *in vitro*	Reference
AGK2	SIRT2	Inhibitor	Increases aerobic glycolysis, OXPHOS, IFN-γ production, and cytotoxic activity in human TILs isolated from NSCLC patient samples	(89)
Thiomyristoyl	SIRT2	Inhibitor	Increases aerobic glycolysis, OXPHOS, and IFN-γ production of human CD3+ T cells from healthy donors	(89)
Sirtinol	Pansirtuin	Inhibitor	Decreases IL8 and TNF (pro-inflammatory cytokines) synthesis in pancreatic cancer cells at the mRNA and protein level	(91)

#### 4.2.2 CD38 and CD157

Multiple myeloma(MM) cells express CD38 in high levels, whereas the expression levels of CD38 are lower in normal myeloid and lymphoid cells as well as some non-hematopoietic tissues, driving the development of anti-CD38 monoclonal antibodies (mAbs) in the treatment of MM. Also, anti-CD38 mAbs have been shown to enhance T cell function and suppress Treg cell proliferation in the tumor microenvironment *in vivo*. In November 2015, Daratumumab, the first human CD38 IgG1κ monoclonal antibody, was approved by the Food and Drug Administration (FDA) as a monotherapy for patients with MM who have received ≥3 prior therapies. Daratumumab has shown significant clinical activity in patients with relapsed or refractory MM when used as either a monotherapy (154) or in combination with lenalidomide and dexamethasone (155). Targeting CD38 by daratumumab induced robust increases in helper and cytotoxic T cell numbers and depleted CD38+ Tregs (156).

Another well-studied CD38 mAb is isatuximab (SAR650894). It induces potent proapoptotic activity in MM cells and elicits complement-dependent cytotoxicity (CDC), antibody-dependent cellular phagocytosis (ADCP), and antibody-dependent cell-mediated cytotoxicity (ADCC) mediated by macrophages (157). Isatuximab also preferentially blocks the induction and function of immunosuppressive CD38^+^ Tregs and restores the immune effector function of NK and CD8+ T effector cells in MM (158). CD38 mAbs, including daratumumab, isatuximab, felzartamab (MOR202), and Ab79 (Millenium/Takeda), show similar mechanisms by binding cells and inducing ADCC. However, they differ in their abilities to inhibit CD38 as well as to mediate apoptosis, ADCP, and especially CDC (159). Other strategies based on the inhibition of CD38 are being developed and evaluated, such as antibody-drug conjugates (ADCs), antibody-recruiting molecules (ARMs), engineered toxin bodies (ETBs), bispecific T-cell engagers (BiTEs) and XmAb Fc domain technology. Many preclinical studies and clinical trials evaluating CD38 inhibition are ongoing or have been completed with patients harboring either hematological malignancies or solid tumors (**Tables 4**, **5**).

**Table 4:** Preclinical studies evaluating the inhibition of CD38 in anticancer immunity.

Type	Name	Effects	Reference
Trispecific T-cell engager	Anti-CD38/CD28 x CD3	Induces the activation and cytokine production by cytotoxic CD8^+^ T cells and the CD38-dependent depletion of autologous primary malignant plasmocytes and immunosuppressive CD38+ MDSCs	(160, 161)
BiTE		Kills a large range of tumor cell lines with superior efficacy than daratumumab	(162)
ETB	TAK-169	Exerts potent cytotoxic effects against CD38-positive human multiple myeloma cell lines *in vitro* and xenograft mouse models	(163)
XmAb	XmAb13243 and XmAb13551 (anti-CD38 x anti-CD3 antibodies)	Stimulates the killing of the RPMI8226human multiple myeloma cell line by human T cells	(164)
XmAb	AMG 424 (anti-CD3 and anti-CD38)	Kills cancer cells and triggers T-cell proliferation *in vitro* Induces tumor growth inhibition in bone marrow-invasive mouse cancer models and the depletion of peripheral B cells in cynomolgus monkeys *in vivo*	(165)
Fc-engineered mAb	SAR442085	Shows higher NK cell-dependent *in vitro* and *in vivo* antimyeloma efficacy than the standards-of-care daratumumab and isatuximab	(166)
Fc multimerization technology	SIFbody	Enhances immunity- and complement-mediated cytotoxicity against tumor cells	(167)
Fc multimerization technology	HexaBody-CD38 (GEN3014)	-Induces greater CDC-mediated tumor cell killing than that induced by daratumumab -Induces FcγR-mediated effector functions and effectively inhibits CD38 enzyme activity, thereby potentially contributing to immune activation	(168)
ADC	STI-6129 (also CD38-077)	Exhibits CD38-dependent cytotoxic activity against a panel of CD38-expressing tumor cell lines	(169)
Radioimmunotherapy	Daratumumab–^225^actinium conjugate	Efficiently and specifically kills CD38^+^ tumor cells *in vitro*	(170)

**Table 5:** Clinical trials to evaluate drug effects on CD38 inhibition in cancer treatments.

Type	Drug	Tumor types	Phase	Clinical trial identifier	Recruitment status
Fc multimerization technology	GEN3014	Relapsed or Refractory Hematologic Malignancies	Phase 1/2	NCT04824794	Recruiting
Fc-engineered mAb	SAR442085	Plasma Cell Myeloma	Phase 1	NCT04000282	Active, not recruiting
ARM	BHV-1100 (Formerly KP1237) + cytokine-induced memory-like (CIML) NK cells	Multiple Myeloma	Phase 1/2	NCT04634435	Recruiting
BiTE	ISB 1342	Relapsed/Refractory Multiple Myeloma	Phase 1	NCT03309111	Recruiting
ADC	STI-6129	Multiple Myeloma	Phase 1/2	NCT05308225	Recruiting
	STI-6129	Relapsed or Refractory Multiple Myeloma	Phase 1/2	NCT05565807	Not yet recruiting
	STI-6129	Advanced Solid Tumor	Phase 1	NCT05584709	Not yet recruiting
	STI-6129	Refractory T Acute Lymphoblastic Leukemia, Acute Myeloid Leukemia (AML)	Phase 1	NCT05519527	Not yet recruiting
XmAb	AMG-424	Relapsed/Refractory Multiple Myeloma	Phase 1	NCT03445663	Terminated
mAb	Isatuximab	Smoldering Plasma Cell Myeloma	Phase 2	NCT02960555	Recruiting
	Isatuximab + atezolizumab	Neoplasms	Phase 1/2	NCT03637764	Terminated
	Isatuximab +Bendamustine +Prednisone	Multiple Myeloma	Phase 1/2	NCT04083898	Recruiting
	Isatuximab +Lenalidomide +Dexamethasone	Plasma Cell Myeloma	Phase 1	NCT01749969	Active, not recruiting
	Isatuximab +Cemiplimab	Plasma Cell Myeloma	Phase 1/2	NCT03194867	Active, not recruiting
	Daratumumab +all-trans retinoic acid (ATRA)	Multiple Myeloma	Phase 1/2	NCT02751255	Active, not recruiting
	Daratumumab /Methylprednisolone /Dexamethasone	Multiple Myeloma	Phase 2	NCT00574288	Completed
	Daratumumab	Plasma Cell Myeloma	Phase 2	NCT02944565	Completed
	Daratumumab	Lymphoma	Phase 2	NCT02927925	Completed
	Daratumumab	Glioblastoma	Phase 1/2	NCT04922723	Recruiting
	Daratumumab	Muscle Invasive Bladder Cancer or Metastatic Kidney Cancer	Early Phase 1	NCT03473730	Active, not recruiting
	Daratumumab	Advanced Prostate Cancer	Phase 1	NCT03177460	Active, not recruiting
	Daratumumab +Atezolizumab	NSCLC	Phase 1/2	NCT03023423	Completed
	Daratumumab +Nivolumab	Advanced Cancer	Phase 1/2	NCT03098550	Completed
	Isatuximab +Pomalidomide +Dexamethasone	Plasma Cell Myeloma	Phase 1	NCT02283775	Completed
	Isatuximab +Cemiplimab	Lymphoma	Phase 1/2	NCT03769181	Completed
	Isatuximab +Cemiplimab	Prostate Cancer, Non-small Cell Lung Cancer	Phase 1/2	NCT03367819	Terminated
	Daratumumab	Multiple Myeloma	Phase 2	NCT03992170	Unknown
	Daratumumab	Multiple Myeloma	Phase 2	NCT03450057	Unknown
	TSK011010 (CID-103)	Multiple Myeloma	Phase 1	NCT04758767	Recruiting
mAb and Fused Protein	TAK-573	Relapsed and/or Refractory Multiple Myeloma	Phase 1	NCT04392648	Withdrawn
	TAK-573 +/Lenalidomide +/Bortezomib +/Carfilzomib +/Daratumumab +/Pomalidomide	Multiple Myeloma	Phase 1	NCT05556616	Recruiting
	TAK-573 +Pembrolizumab	Advanced or Metastatic Solid Tumors	Phase 1/2	NCT04157517	Recruiting
	TAK-573 +Dexamethasone	Multiple Myeloma	Phase 1/2	NCT03215030	Recruiting
	TAK-573 +Daratumumab	Multiple Myeloma	Phase 1/2	NCT05590377	Recruiting

Furthermore, CD38, as a NADase, regulates a wide range of NAD-dependent cellular processes as mentioned above. Although most anti-CD38 mAbs have been reported to mainly function via their cytolytic effects, isatuximab and daratumumab also inhibit enzymatic activities of CD38 to some extent (154, 171, 172). Their therapeutic effects are unknown related to inhibition of CD38 enzymatic activity as well. In fact, given the role of CD38 metabolism in solid tumor microenvironment, metabolism reprogramming of NAD^+^ regulation via CD38 inhibition has attracted attention as a strategy for immunotherapy, either with anti-CD38 antibodies alone or in combination with immunomodulatory drugs. For instance, in a preclinical melanoma model, Chatterjee et al. demonstrated that blocking CD38 expression on T cells boosts NAD^+^ levels and improves the efficacy of adoptive transferred T cells (173). Moreover, targeting the eADO pathway can increase the antitumor effects of drugs through various mechanisms, such as by enhancing T-cell and natural killer cell functions, promoting antigen presentation, and inhibiting the immunosuppressive effects of MDSC (104). In particular, we identified CD73, a nucleotidase in the eADO pathway, as an immunotherapy response predictor and prognostic biomarker in head and neck squamous cell carcinoma (174). Similarly, CD38, which also participates in the adenosinergic pathway, has been reported to be an effective predictor of anti-PD-1 antibody-based checkpoint immunotherapy responses in hepatocellular carcinoma (175).

In addition, apart from monoclonal antibodies, there are currently more than 200 small-molecule inhibitors that suppress the enzymatic activity of CD38 (176). Although these small molecule inhibitors of CD38 have not been reported in the literature for their use in tumor treatment, we strongly believe that these inhibitors will at least become powerful tools for unveiling the role of the enzymatic function of CD38 in tumors and solid TME.

Finally, although the regulatory mechanism underlying CD157 effects in the immunosuppressive TME remains unclear, an Fc-optimized antibody against BST1/CD157, MEN1112/OBT357NF, exhibited effective ADCC *in vitro* (177). A Phase 1 clinical trial to evaluate MEN1112 in patients with relapsed or refractory acute myeloid leukemia (NCT02353143) is ongoing.

## 5 Conclusions

In summary, the enzymes involved in NAD^+^ metabolism are extensively involved in various intracellular signaling pathways and play a nonnegligible role in the development of immunosuppressive TME.

On the one hand, upregulated NAD^+^ anabolism contributes to metabolic reprogramming, the EMT, cell stemness, etc. Pharmacological inhibition of key enzymes involved in NAD^+^ production, mainly NAMPTis and a few NAPRTis, that impair NAD^+^ generation has exhibited promising results in cancer cell lines and preclinical studies. Unfortunately, NAMPTi monotherapy has been proven to be limited in clinical trials. Considering that different tumors vary in their dependence on the NAD^+^ synthesis pathway, the development of only NAMPT inhibitors has been an insufficient approach to date. Potent-specific NAPRT inhibitors need to also be developed. Selecting NAMPTis or NAPRTis according to the dependence of cancers on NAD^+^ synthesis may further increase the effectiveness of targeted NAD^+^ synthesis therapy. In addition, extracellular NAMPT and NAPRT are involved in regulating the immunosuppressive TME; therefore, specifically targeting extracellular NAMPTis and NAPRTis may increase the effectiveness of NAD^+^ inhibition therapy and potentially enhance the efficacy of immunotherapy.

In addition, NAD-degrading enzymes, especially CD38, play critical roles in tumor cell immune evasion via the adenosinergic pathway. CD38 inhibition has attracted attention as a strategy for immunotherapy, either with anti-CD38 antibodies alone or in combination with other immunomodulatory drugs. Indeed, many preclinical studies and clinical trials to evaluate CD38 inhibition in both solid tumors and hematological malignancies are ongoing or have been completed. Targeting CD38 is considered to have been a breakthrough in multiple myeloma immunotherapies. But from another perspective, there are still many unresolved issues in the future application of anti-CD38 monoclonal antibodies. The first is whether, in addition to isatuximab and daratumumab, other anti-CD38 monoclonal antibodies inhibit CD38 enzymatic activity. Also, the impact of CD38 enzymatic activity on the immune system and tumor microenvironment still needs to be elucidated, besides participating in adenosine pathway. Secondly, the CD38 monoclonal antibody inhibits the extracellular NADase activity of CD38 and increases the level of extracellular NAD^+^. On the other hand, the increase in intracellular NAD^+^ is beneficial to tumor growth. Therefore, it still needs to be clarified whether the CD38 monoclonal antibody leads to an increase in the intracellular NAD^+^ level and whether it plays a double-edged sword role in tumor treatment. Finally, small molecule inhibitors of CD38 can inhibit the enzymatic activity of CD38. Using these tools, we can better understand the role of CD38 enzymatic activity in tumors. Also, apart from CD38, other NADases may also promote tumor cell immune escape, but to understand the specific role they play, further research is still needed.

## Author contributions

JY: Conceptualization, Resources, Writing – original draft, Writing – review & editing. SC: Resources, Visualization, Writing – review & editing. ZZ: Conceptualization, Funding acquisition, Supervision, Writing – review & editing.
